# Letter to the Editor regarding the Article: Glenosphere Size Does Not Matter in Reverse Total Shoulder Arthroplasty. Rev Bras Ortop. 2024;59(2):254–259

**DOI:** 10.1055/s-0044-1800943

**Published:** 2025-03-04

**Authors:** João Artur Bonadiman

**Affiliations:** 1 Instituto de Ortopedia e Traumatologia, Hospital São Vicente de Paulo, Passo Fundo, RS, Brasil.; 2 Instituto Brasil de Tecnologias da Saúde, Rio de Janeiro, RJ, Brasil.

With great interest, I read the article: “Glenosphere Size Does Not Matter in Reverse Total Shoulder Arthroplasty” published by Patel et al. 1 Studies focusing on the clinical outcomes of reverse total shoulder arthroplasty (RTSA) are always important given the linear growth and “popularization” of this procedure. Given the conclusion of the published study (which was the basis for its title), it is necessary to scrutinize the details of the applied methodology, due to the impact and significance of its repercussions. Some details could help us understand the outcomes obtained in such research, especially regarding the surgical technique, rehabilitation, and chronology of the applied methodology.

## Surgical Technique and Rehabilitation

Considering the wide range of configurations available for the design of RTSA (such as inlay versus onlay, lateralization of the glenosphere, neck-shaft angle, tilt, among others) and their respective influences on functional outcomes, 2 3 a detailed understanding of the specific type of implant used in the study under analysis is crucial. The precise identification of the prosthesis used is fundamental for replicating the results, allowing for a more accurate assessment of the efficacy of the applied technique.

The reinsertion or not of the subscapularis tendon in RTSA is a widely discussed factor that has significant impacts, especially regarding the range of motion. The elucidation of these data in the article will provide readers with a solid foundation to assess the methodologies and outcomes of the study. 4

Regarding the rehabilitation process: there is a lack of precise information about which protocol was adopted postsurgery, as well as the immobilization period with a sling, which type of sling was used, and when physiotherapeutic rehabilitation was initiated. The rehabilitation protocols after reverse arthroplasty are often discussed and have a significant impact on the final degree of mobility. 5

## Chronology

The study, developed at the Department of Orthopedic Surgery, Icahn School of Medicine at Mount Sinai, New York, United States, mentions that patients were included who underwent reverse shoulder arthroplasty since 1987. However, such a procedure was approved by the Food and Drug Administration 16 years later, in November 2003 (Delta Shoulder K021478; DePuy Inc., Raynham, MA, USA). 6 I believe that clarifying this chronological discrepancy is essential for a proper understanding of the study's methodology.

The range of motion—which is the most important aspect of the study in question—was measured by being divided, pre- and postoperatively. However, in the study, it is not clear at what point the postoperative measurement was taken. The precise determination of this interval is crucial, as it directly influences the interpretation of the results and the validity of the study.

The simple shoulder test (SST) score was applied for pre- and postoperative evaluation of patients. However, there is a methodological gap regarding the data collection of patients who underwent surgery before 1993, the year in which the SST was formally described. 7

In summary, it is imperative to emphasize that the detailed deepening of the information presented is crucial, considering the substantial relevance of the findings reported in this study and the significant potential impact of this publication in the scientific field.
