# Supplementary material for: Letter to the Editor regarding the Article: Glenosphere Size Does Not Matter in Reverse Total Shoulder Arthroplasty. Rev Bras Ortop. 2024;59(2):254–259
Source: Rev Bras Ortop (Sao Paulo). 2025 Mar 4;60(1):s00441800943. doi: 10.1055/s-0044-1800943 (PMC11879487; doi:10.1055/s-0044-1800943)
Supplement: Related texts

# Research article: Carta ao editor sobre o artigo: O tamanho da glenosfera não importa na artroplastia total reversa de ombro. Rev Bras Ortop. 2024;59(2):254–259

Com grande interesse, li o artigo “O tamanho da glenosfera não importa na artroplastia total reversa de ombro” publicado por Patel et al. 1 Estudos com foco nos resultados clínicos da artroplastia total reversa do ombro (ATR) são sempre importantes devido ao crescimento linear e a “popularização” deste procedimento. Dada a conclusão do estudo publicado (que foi a base para seu título), é necessário escrutinar os detalhes da metodologia aplicada devido ao impacto e à importância de suas repercussões. Alguns detalhes podem nos ajudar a entender os resultados obtidos em tal pesquisa, em especial aqueles referentes à técnica cirúrgica, reabilitação e cronologia da metodologia aplicada.

## Técnica Cirúrgica e Reabilitação

Considerando a ampla gama de configurações disponíveis para a ATR (como *inlay* versus *onlay* , lateralização da glenosfera, ângulo colo-diáfise, inclinação, entre outras) e suas respectivas influências nos resultados funcionais, 2 3 torna-se crucial compreender de maneira detalhada o tipo específico de implante utilizado no estudo em análise. A identificação precisa da prótese utilizada é fundamental para replicar os resultados, permitindo uma avaliação mais precisa da eficácia da técnica aplicada.

A reinserção ou não do tendão subescapular na ATR é um fator amplamente discutido e que tem impactos significativos, principalmente no que se refere à amplitude de movimento. A elucidação desses dados no artigo fornecerá aos leitores uma base sólida para avaliar as metodologias e os resultados do estudo. 4

Quanto ao processo de reabilitação, faltam informações precisas sobre qual protocolo foi adotado no pós-operatório, bem como o período de imobilização com tipoia, qual tipo de tipoia foi utilizado e o momento de início da reabilitação fisioterapêutica. Os protocolos de reabilitação após a ATR são frequentemente discutidos e têm um impacto significativo no grau de mobilidade final. 5

## Cronologia

O estudo, desenvolvido no Departamento de Cirurgia Ortopédica, Icahn School of Medicine at Mount Sinai, em Nova York, Estados Unidos, menciona que foram incluídos pacientes submetidos à ATR desde 1987. No entanto, tal procedimento foi aprovado pela *Food and Drug Administration* 16 anos depois, em novembro de 2003 (Delta Shoulder K021478; DePuy Inc., Raynham, MA, EUA). 6 Acredito que esclarecer essa discrepância cronológica é essencial para uma compreensão adequada da metodologia do estudo.

A amplitude de movimento – o aspecto mais importante do estudo em questão – foi medida e dividida entre pré- e pós-operatória. No entanto, o estudo não deixa claro em que ponto a medição pós-operatória foi feita. A determinação precisa desse intervalo é crucial, pois influencia diretamente a interpretação dos resultados e a validade do estudo.

O escore do Simple Shoulder Test (SST) foi aplicado para avaliação pré- e pós-operatória dos pacientes. Entretanto, há uma lacuna metodológica quanto à coleta de dados de pacientes submetidos à cirurgia antes de 1993, ano em que o SST foi formalmente descrito. 7

Em suma, é imprescindível ressaltar que o aprofundamento detalhado das informações apresentadas é fundamental, tendo em vista a relevância substancial dos achados relatados neste estudo e o significativo potencial de impacto desta publicação no campo científico.
